# Endoscopic management of ureteral injuries arising from gynecologic procedures

**DOI:** 10.1002/bco2.70160

**Published:** 2026-02-11

**Authors:** Ari Luder, Asaf Shvero, Roey Mashiach, Zohar A. Dotan, Nir Kleinmann

**Affiliations:** ^1^ The Department of Urology Sheba Medical Center Ramat Gan Israel; ^2^ Affiliated With the Faculty of Medicine Tel Aviv University Tel Aviv Israel; ^3^ The Department of Obstetrics and Gynecology Sheba Medical Center Ramat Gan Israel

**Keywords:** gynecologic surgery, iatrogenic complications, retrograde endoscopic management, ureteral injury, ureteral stricture

## Abstract

**Introduction:**

Ureteral injuries during gynecologic surgery are uncommon (0.1%–2.5%) but may result in significant morbidity, including strictures, hydronephrosis and the need for additional interventions. This study evaluates the effectiveness of retrograde endoscopic management in treating iatrogenic ureteral injuries.

**Materials and Methods:**

A retrospective review was conducted on women diagnosed with ureteral injury post‐gynecologic surgery between 2010 and 2024 at a single institution. Patients were categorized into two groups: those treated with retrograde endoscopic interventions and those managed with non‐endoscopic approaches (percutaneous nephrostomy and/or surgical reconstruction). The endoscopic group was further divided into early (<3 months post‐injury) and late interventions. Outcomes assessed at ≥3 months of follow‐up included treatment success, long‐term complications and the need for further interventions.

**Results:**

Of 42 patients, 29 (69%) underwent endoscopic treatment and 13 (31%) received non‐endoscopic management. Among endoscopically treated patients, early intervention achieved an 80% success rate, significantly higher than the 33% observed with late intervention (*p* = 0.03). All non‐endoscopic patients initially received percutaneous nephrostomy, and 12 (92.3%) required definitive surgical repair. Endoscopic treatment was associated with reduced operative time and shorter hospital stays. Given the rarity of ureteral injuries, the cohort represents one of the largest single‐centre experiences focused on this specific population.

**Conclusions:**

Early retrograde endoscopic management is a safe and effective approach for treating ureteral injuries after gynecologic surgery. Timely diagnosis and intervention significantly improve outcomes. Non‐endoscopic patients were more complex cases, often unsuitable for endoscopy, which may account for outcome differences. Intraoperative retrograde ureterography and stenting should be considered whenever there is suspicion of ureteric injury, whereas postoperative endoscopic realignment or endoscopic management of ureteric strictures should be performed by appropriately trained urologists. Further prospective studies with larger cohorts and longer follow‐up are warranted to refine optimal clinical pathways and long‐term management strategies.

## 1 INTRODUCTION

Ureteral injuries during gynecologic surgeries are rare but have potentially serious consequences. These injuries are well‐recognized complications of gynecologic and obstetric surgeries due to the anatomical proximity of the internal female reproductive organs and the urinary tract. Distal ureteral injuries represent the most common site of injury, accounting for 91% of ureteral injuries in gynaecological surgeries.^1^ The risk of urological injuries varies by procedure and surgical method. A systematic review of 90 studies involving 140 444 gynecologic surgeries between 1975 and 2015 reported an incidence of ureteral and bladder injuries during laparoscopy for benign conditions of 0.24%. The incidence was 0.44% during Caesarean sections and 1.54% during hysterectomies for benign indications.^2^

Risk factors for ureteral injury include prior pelvic or abdominal surgeries, adhesions, endometriosis and anatomical anomalies.^3^ These factors complicate ureteral identification and increase the risk of iatrogenic injury. Consequences of such injuries include ureteral leaks, ureteral strictures, hydronephrosis and the need for further interventions, such as urinary diversion or ureteral reconstruction.^4^

Timely intervention (in one study, ideally between 1‐ and 42‐day post‐injury) has been shown to reduce complications and improve outcomes. In a study of 23 patients, 30% required auxiliary procedures for ureteral injuries. Six patients developed vesicovaginal fistulas, and five experienced severe complications, including recurrent urinary tract infections (UTIs) and mucosal excoriation. The authors emphasized that intraoperative recognition and prompt management of ureteral injuries significantly reduce the risk of long‐term complications.^5^

**FIGURE 1:**
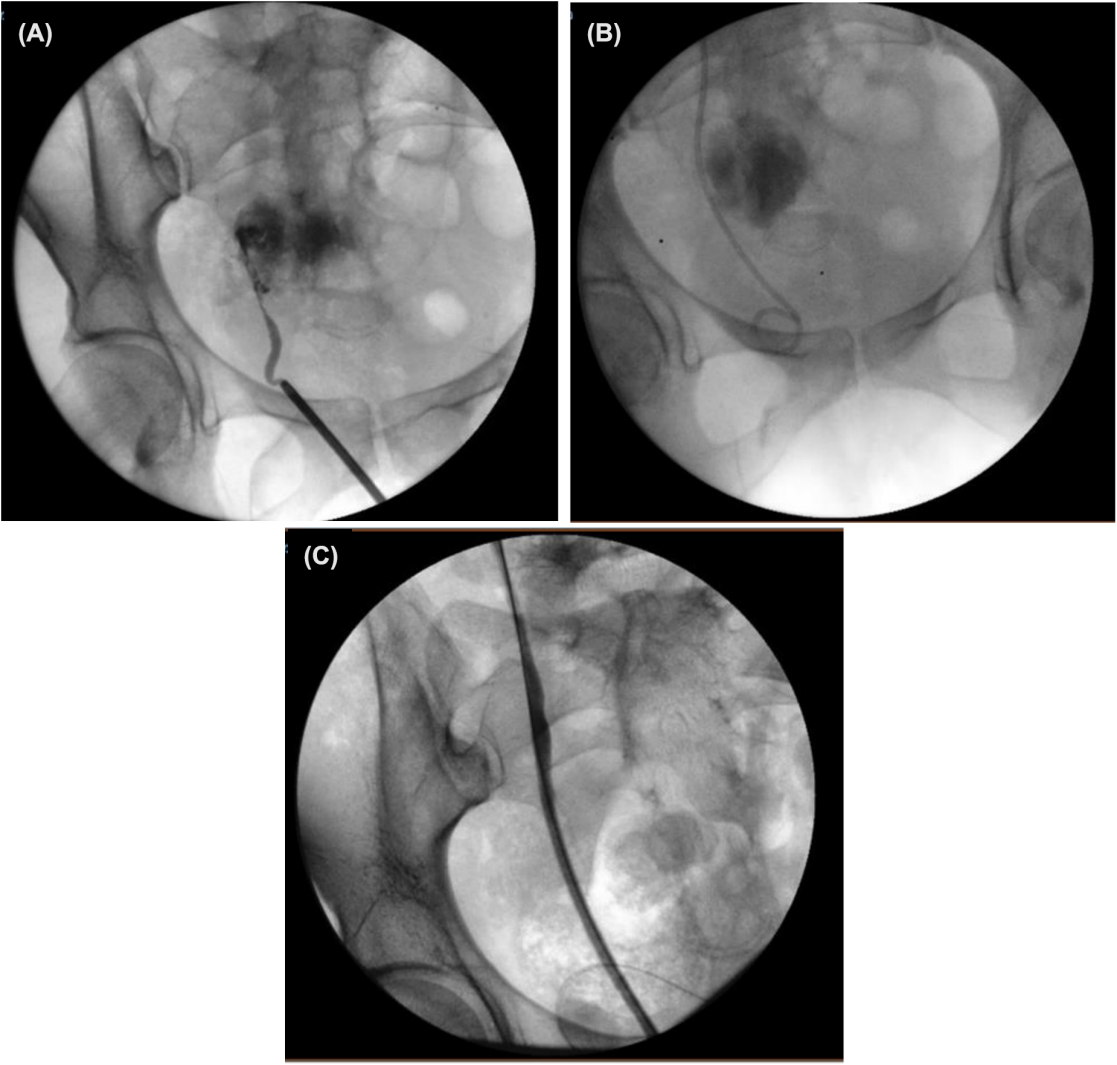
(A) Retrograde pyelogram demonstrates a complete transection of the right distal ureter, with no passage of contrast observed. (B) Initial ureteral recalibration and double‐J stent placement. (C) On follow‐up 6 weeks after stent insertion, full recovery was observed with no evidence of a ureteral stricture.

Retrograde endoscopic treatment has emerged as a valuable intervention for managing ureteral injuries, providing a minimally invasive option for ureteral recalibration and stent insertion.^6^ However, data on its efficacy and long‐term outcomes remain limited. This study aimed to evaluate the feasibility and efficacy of retrograde endoscopic treatment for iatrogenic ureteral injuries (Figure 1).

## 2 METHODS

We conducted a retrospective cohort study of patients diagnosed with iatrogenic ureteral injuries following gynecologic surgery between 2010 and 2024. Eligible patients were identified using institutional databases. Inclusion criteria consisted of radiologically or endoscopically confirmed ureteral injury directly attributable to a gynecologic procedure. Patients with pre‐existing ureteral pathology (including prior ureteral strictures, prior ureteric or ureteroscopic interventions for urological conditions such as lithiasis, congenital ureteral anomalies or chronic kidney disease with eGFR <45 mL/min/1.73 m^2^), as well as those with less than 6 months of follow‐up, were excluded to minimize selection bias and ensure a homogeneous study population.

Participants were grouped according to treatment modality: those managed with retrograde endoscopic treatments and those treated with non‐endoscopic methods, including percutaneous nephrostomy and/or primary reconstructive surgery (e.g., ureteroureterostomy and ureteral reimplantation). Patients were allocated to treatment groups based on clinical presentation and feasibility of endoscopic management. Those with complete transections or delayed diagnosis often required non‐endoscopic management. The endoscopic group was further subdivided into early treatment (within 3 months of injury) and late treatment (beyond 3 months). We compared the endoscopic group with the non‐endoscopic group for baseline characteristics and outcomes and the same for the early versus late endoscopic subgroups.

In our cohort, the choice between endoscopic and non‐endoscopic management was not the result of an intentional, predefined clinical selection process but rather reflected the timing and clinical status at presentation. All women in the study initially underwent gynecologic surgery and were treated postoperatively in gynaecology units. None were identified perioperatively as having a ureteral injury, and therefore no early urologic triage or decision‐making regarding endoscopic versus non‐endoscopic treatment occurred at that stage. After discharge from the gynaecology service, patients presented to urologic evaluation only when they became symptomatic, most commonly with flank pain, hydronephrosis on imaging, acute kidney injury, urinary leakage or sepsis. Women presenting early and in a stable condition were generally treated with retrograde stenting or other endoscopic methods, provided that retrograde access was feasible. In contrast, women presenting late, in sepsis, with significant urinoma, or when retrograde access was not possible, were treated with percutaneous nephrostomy (non‐endoscopic drainage) as the safest and most urgent intervention. Thus, the management pathway in this cohort reflects a real‐world scenario of missed perioperative ureteral injuries, in which the ‘choice’ of treatment is dictated by clinical urgency and anatomical feasibility at delayed presentation rather than by a predetermined algorithm.

Primary endpoints were long‐term (≥3 months) complications, including ureteral strictures, urinary leakage and renal function deterioration. Long‐term outcomes were defined as events occurring at least 3 months after definitive intervention; where available, outcomes at approximately 12 months of follow‐up were also recorded and descriptively analysed. Secondary outcomes included the need for surgical reconstruction and stent dependency. Treatment success was defined as a resolution without reconstructive surgery or permanent stent dependency.

Within the endoscopic groups, we compared outcomes between patients receiving 6Fr and 7Fr stents and between those treated early versus late

### 2.1 Endoscopic Technique

Endoscopic interventions were performed under general anaesthesia by fellowship‐trained endourologists. The procedure included retrograde ureteropyelography to demonstrate the location and extent of the injury, semirigid ureteroscopy (when direct vision was needed), balloon dilation and fluoroscopy‐guided ureteral stent placement. Stent selection varied by calibre (6Fr or 7Fr), length (24–26 cm) and configuration (single or tandem), chosen based on intraoperative findings.

### 2.2 Follow‐up protocol

Patients were followed up in the urologic clinic. The endoscopic group was followed monthly or bi‐monthly with clinical evaluation, renal ultrasound and kidney function tests. Based on findings and the extent of the injury, patients either had their stent removed in the office or were taken to the OR for retrograde evaluation and either had their stent removed in the OR or underwent a stent exchange and were referred for reconstructive surgery. The non‐endoscopic group was followed post‐reconstructive surgery with similar assessments. Postoperative complications were recorded and classified according to the Clavien–Dindo grading system for descriptive purposes.

### 2.3 Statistical analysis

All statistical analyses were performed using SPSS v26.0 (IBM Corp.). Categorical variables were compared with chi‐square or Fisher's exact tests, and continuous variables with Mann–Whitney *U* tests. Statistical significance was set at *p* < 0.05. Exploratory analyses were conducted to evaluate whether outcomes varied by mechanism of injury (electrocautery, vigorous dissection or ligation); however, these comparisons were underpowered and are interpreted cautiously.

## 3 RESULTS

Demographic and surgical characteristics are shown in Table 1. The cohort included 42 women with a mean age of 45.8 years. The endoscopic (*n* = 29) and non‐endoscopic (*n* = 13) groups were comparable in age, BMI, surgical modality of the gynecologic surgery (open vs. laparoscopic), mechanism of injury and location of injury. Baseline characteristics were similar between groups, but non‐endoscopic patients more frequently presented with severe injuries or late diagnoses, explaining the higher need for reconstruction. The distal ureter was the most commonly affected site (64.3% of cases).

**TABLE 1:** Demographics and clinical characteristics of participants.

Variables	Total (*n* = 42)	Study group		*p*‐value
		Endoscopic Treatment (*n* = 29)	Non‐endoscopic treatment (*n* = 13)	
Age, years	42 (100)	45 (69.04)	46.84 (30.96)	0.785
BMI	42 (100)	25.6 (69.04)	25.30 (30.96)	0.555
Type of gynaecological surgery, *n* (%)				0.150
Hysterectomy	23 (54.76)	18 (62.06)	5 (38.46)	
Debridement	4 (9.52)	2 (6.89)	2 (15.38)	
Endometrioma resection	3 (7.14)	3 (10.34)	0 (0.00)	
Resection of pelvic mass	4 (9.52)	1 (3.44)	3 (23.07)	
Other	8 (19.04)	5 (17.24)	3 (23.07)	
Modality associated with injury, *n* (%)	42 (100.0)			0.232
Open	22 (52.38)	17 (58.62)	5 (38.46)	
Laparoscopic	20 (47.62)	12 (41.37)	8 (61.53)	
Side of ureter injury, *n* (%)	42 (100)			0.937
Right ureter	19 (45.2)	13 (44.83)	6 (46.15)	
Left ureter	23 (54.8)	16 (55.17)	7 (53.85)	
Extent of injury, *n* (%)				0.348
Partial	30 (71.32)	22 (75.86)	8 (61.53)	
Complete	12 (28.68)	7 (24.14)	5 (38.47)	
Location of ureter Injury, *n* (%)				0.214
Middle ureter	9 (21.41)	4 (13.79)	5 (38.46)	
Distal ureter	27 (64.30)	20 (68.96)	7 (53.84)	
Multiple	6 (14.29)	5 (17.24)	1 (7.69)	
Average injury length (cm) ± SD				
		1.69 (0.35)	1.77 (0.35)	0.214
Mechanism of injury, *n* (%)				0.254
Electrocautery	9 (21.41)	8 (27.58)	1 (7.69)	
Vigorous dissection	27 (64.28)	18 (62.06)	9 (69.23)	
Ligation	6 (14.28)	3 (10.34)	3 (23.07)	

### 3.1 Endoscopic vs. non‐endoscopic management

Treatment success, defined as resolution without reconstructive surgery or long‐term stenting, was achieved in 69% of patients in the endoscopic group. The median interval between injury and definitive intervention was significantly shorter among patients in the endoscopic group compared to those in the non‐endoscopic group (1.72 vs 8.61 months; *p* = 0.008). Long‐term outcomes were assessed at ≥3 months after the definitive intervention in all included patients.

Impact of timing of endoscopic treatment on outcomes is shown in Table 2. Patients treated within 3‐month post‐injury (*n* = 20) had significantly better outcomes than those treated later (*n* = 9). Early intervention resulted in a higher treatment success rate (80% vs. 33.3%, *p* = 0.0317) and lower stricture rate (20% vs. 66.6%, *p* = 0.032). Delayed diagnosis (>3‐month post‐injury) led to ureteral stent dependency or surgical reconstruction in 77.7% (7 out of 9 patients).

**TABLE 2:** Comparison of complication rates by timing of endoscopic management.

Variables	Treated <3 months post injury (*n* = 20)	Treated >3 months after injury (*n* = 9)	*p*‐value
Mean time (days)	35	129	
Long‐term Complications, *n* (%)			0.0317
Renal failure	0 (0.00%)	0 (0.00%)	
Ureteral leak	0 (0.00%)	0 (0.00%)	
Ureteral stricture	4 (20%)	6 (66.6%)	
Treatment success (no stent dependency)	16 (80%)	3 (33.3%)	

Among patients who underwent endoscopic treatment with single stents (Table 3), 30% of those with 6Fr stents had long‐term complications (10% decline in renal function and 30% ureteral strictures). In comparison, 14.3% of patients in the 7Fr group developed complications—7.1% with ureteral strictures and 7.1% with ureteral leakage—with no observed decline in renal function (*p* = 0.192).

**TABLE 3:** Long‐term outcomes of endoscopic management using 6Fr vs. 7Fr double‐J ureteral stents: A comparative analysis.

Variables	Single stent 6Fr (*n* = 10)	Single stent 7Fr (*n* = 14)	*p*‐value
Long‐term complications, *n* (%)			0.192
None	6 (60.0%)	12 (85.7%)	
Renal failure	1 (10.0%)	0 (0.00%)	
Ureteral leak	0 (0.00%)	1 (7.1%)	
Ureteral stricture	3 (30.0%)	1 (7.1%)	

Surgical reconstruction was needed in 92.3% of the non‐endoscopic group and in 24.1% of cases in the endoscopic group (initially managed endoscopically). Operative and perioperative data on the reconstructive surgeries are shown in Table 4. Length‐of‐stay (LOS) was shorter in patients who were initially managed endoscopically (5.4 vs. 6.2 days, *p* = 0.33) as was the duration of surgery (121.5 vs. 135.2 min, *p* = 0.018). Endoscopic procedures were significantly shorter than reconstructive surgeries (30 vs. 135.2 min, *p* = 0.001) with shorter LOS (1.78 vs. 5.3 days; *p* = 0.0008).

**TABLE 4:** Operative and perioperative data of cases undergoing surgical reconstruction.

Variables	Non‐endoscopic group (*n* = 13)	Patients managed initially endoscopically (*n* = 7)	*p*‐value
Number of patients	13	7	
Type of surgery			
Ureteral reimplantation	8	2	
Ureteroureterostomy	4	5	
Percutaneous Nephrostomy	1		
Operative time minutes (IQR)	135.2 (8)	121.5 (5)	0.018
Length‐of‐stay days (IQR)	6.2 (1.3)	5.4 (1.4)	0.33
Treatment success (no stent dependency)	12 (92.3%)	6 (85.7%)	0.9

In exploratory analyses, outcomes did not demonstrate a clear pattern of variation by mechanism of injury (electrocautery, vigorous dissection or ligation), although the study was not powered to detect modest differences between these subgroups.

## 4 DISCUSSION

Iatrogenic ureteral injuries sustained during gynecologic surgeries are a well‐known complication with significant sequelae, with an incidence of 0.3%–1.8%.^1^, ^2^ When identified during surgery, these injuries are usually either primarily repaired or managed with ureteral stent insertion, which yields favourable outcomes.^7^, ^8^ However, in 87% of cases, these injuries are left unrecognized initially, later presenting with flank pain, UTIs or acute kidney injury.^3^, ^4^ These cases are challenging, with risks of sepsis, urinoma, fistula and ureteral strictures.^7^, ^8^, ^9^, ^10^ Until recently, the only option was an invasive reconstructive surgery. Due to recent advances in endoscopic methods, a retrograde approach became more appealing, providing a minimally invasive treatment option for a wide variety of urological conditions. We set out to assess the role of retrograde endoscopic interventions in managing these cases of ureteral injuries diagnosed after gynaecological surgeries.

Our study shows the efficacy of early retrograde endoscopic interventions in managing these iatrogenic ureteral injuries. Early diagnosis and treatment within 3 months of injury resulted in an 80% treatment success rate. Timing of the endoscopic intervention plays a critical role in treatment success. Patients treated later than 3 months after injury had a significantly lower treatment success rate (33.3%) compared to patients treated early (80%, *p* = 0.03). The progressive fibrotic remodelling and chronic inflammation resulting from delayed intervention, often culminating in irreversible damage that mandates surgical reconstruction,^11^ might explain these findings. Patients who failed the endoscopic approach remained with a ureteral stricture and were stent‐dependent until a reconstructive surgery was pursued.

We also explored the role of stent calibre in treatment success, a subject with limited existing data. We theorized that larger‐calibre stents might have potentially better outcomes due to improved drainage and better lumen integrity. This observation complements the hypothesis presented by Miller et al., who suggested that stent calibre may influence ureteral patency and healing rates.^12^ Despite that, the rates of success we found in our cohort were comparable between 6Fr and 7Fr stents (two of the largest groups of stents with 10 and 14 patients, respectively). Other studies demonstrated that ureteral stent calibre does not significantly influence complication rates.^9^, ^13^ As suggested by others, it seems that the timing of the intervention outweighs stent design in affecting outcome.

Patients treated with non‐endoscopic modalities underwent an initial percutaneous nephrostomy insertion, an antegrade contrast study and a majority ultimately underwent surgical reconstruction (92.3%). Although reconstructive surgeries were successful in 90%, these represent a surgical challenge, with a duration range from 120 to 180 min and length‐of‐stay (LOS) of 6.24 days. Among patients who underwent an initial endoscopic management with a following reconstructive surgery (7 patients), the surgical duration of the reconstructive surgery was 121.5 min, and LOS was 5.4 days. Compared to reconstructive surgeries, endoscopic treatments had a much shorter duration (30 min, *p* = 0.001) and a much shorter LOS (1–2 days, *p* = 0.0008).^14^ These differences highlight the benefits of this minimally invasive treatment, when feasible. These findings reflect the outcomes reported by Garcia et al.,^15^ and Wright et al.,^16^ who demonstrated increased morbidity and prolonged recovery times in surgically managed ureteral injuries compared to those managed endoscopically. Due to the high success rate of endoscopic treatments, it seems reasonable to initially attempt such treatment, with reconstructive surgery reserved for cases with persistent ureteral strictures and stent dependency after the endoscopic treatment.^17^

Our results are consistent with prior series evaluating endoscopic management of iatrogenic ureteral injuries, which have reported favourable success rates and reduced morbidity compared with open reconstruction in selected patients.^16^, ^17^ Similar to the multicentre study by Cestari et al.,^17^ we found that endoscopic management can obviate or defer major reconstructive surgery in a substantial proportion of patients, particularly when intervention is undertaken early. Moreover, the shorter operative times and hospital stays observed in our cohort mirror previously reported advantages of minimally invasive approaches in urology.^14^, ^15^, ^16^

The worse outcomes in the non‐endoscopic group reflect inherent case severity and not solely treatment modality. This selection bias limits direct comparison but underscores the importance of early diagnosis, when endoscopic management remains feasible. Despite a relatively small number of cases, this reflects the rarity of the condition. The current cohort is one of the largest in the literature examining endoscopic management in this context. In addition, although we explored outcomes according to mechanism of injury (electrocautery, dissection and ligation), we did not observe a clear difference in success or complication rates between these subgroups; however, the small numbers within each category preclude definitive conclusions.

The current study contributes novel insights in key aspects, but it is not devoid of limitations. First, it is retrospective in nature, which exposes it to selection bias. Second, the sample size for this fortunately rare condition is relatively low and limits statistical analyses. Third, although all patients had at least 6 months of follow‐up and long‐term outcomes were assessed at ≥3 months after definitive intervention, the duration of follow‐up varied and may not fully capture very late complications, such as delayed strictures beyond the first postoperative year. Longer and more uniform follow‐up in larger, prospective cohorts would help to better define the true long‐term efficacy of endoscopic management in this setting. Lastly, our centre is a high‐volume tertiary centre, and our results may not be generalized to lower volume institutions or nonspecialist settings. Despite these limitations, our study represents one of the largest focused cohorts to evaluate the impact of treatment timing, stent configuration and procedural modality in gynecologic‐associated ureteral injuries.

## 5 CONCLUSIONS

Endoscopic treatments for iatrogenic ureteral injuries sustained during gynecologic surgery represent a valid and effective therapeutic option. Early retrograde stent placement significantly reduces the incidence of ureteral strictures and the need for invasive reconstructive procedures. Given these advantages, institutional protocols should prioritize prompt retrograde assessment and stenting whenever ureteric injury is suspected. Intraoperative retrograde ureterography and stenting can often be performed by the attending surgeon or an on call urologist, whereas postoperative endoscopic realignment or endoscopic management of ureteric strictures should be undertaken by urologists with appropriate endourologic training and experience. Our findings support early retrograde endoscopy as the first‐line intervention in eligible cases, while recognizing that complex or severe injuries will still necessitate definitive reconstructive surgery.

## AUTHOR CONTRIBUTIONS

**Ari Luder:** Conceptualization, study design, data interpretation, primary manuscript writing. **Asaf Shvero:** Manuscript editing, critical revision. **Roey Mashiach:** Data collection. **Zohar A. Dotan:** Senior review, clinical supervision. **Nir Kleinmann:** Data gathering, topic supervision, academic instruction.

## CONFLICT OF INTEREST STATEMENT

The authors declare no conflicts of interest.
